# Comments on “Splitting tensile strength prediction of sustainable high-performance concrete using machine learning techniques” by Wu, Yangi et al., https://doi.org/10.1007/s11356-022-22048-2

**DOI:** 10.1007/s11356-023-28829-7

**Published:** 2023-07-21

**Authors:** Ozgur Kisi, Sara Ajri, Kim Cedric Jörgens, Arti Karande, Sabine Kraus, Benita Naumann, Kim Nierman, Wiebke Seel, Christoph Kulls

**Affiliations:** 1 Department of Civil Engineering, Lübeck University of Applied Sciences, 23562 Lübeck, Germany; 2 Department of Civil Engineering, Ilia State University, 0162 Tbilisi, Georgia

The discussers would like to thank the authors for investigating the efficiency of machine learning methods in predicting splitting tensile strength of high-performance concrete (HPC). In the discussed study (Wu and Zhou 2022), two hybrid genetic algorithm (GA)-based artificial neural networks (ANN) and grid search (GS)-based support vector regression are compared with classical ANN using empirical data collected from published literature and they conclude that the GS-SVR performance is superior to the other methods in predicting splitting tensile strength of HPC. The discussers would like to raise some important issues, which can be taken into account by the authors for future studies and audiences.

In the study of (Wu and Zhou 2022), the parameter settings of the GA-ANN have been provided in Table 1 (see Table 2 in the original study). In this table trainlm is a MATLAB command indicating the Levenberg–Marquardt (LM) algorithm which is used for training classical ANN. We wonder how the authors could use this algorithm in training while they also state that they calibrated ANN using GA which is an evolutionary method and that does not use LM. Furthermore, there is no information about cross-over or mutation operators and their values/settings which are very important in GA optimization. How did you find the optimal settings for the GA-ANN model? If you used a trial and error approach, which method did you follow? Did you optimize the hidden node number by setting generation and population size constant or was another procedure applied? These open questions need further explanation and do not make the GA-ANN model reproducible. On the other hand, there is not enough information about the classical ANN model (e.g., iteration number, activation functions, hidden node number) developed in Wu and Zhou (2022). Which software or program did the authors use for models simulations? Where is the parameters of GS and how did you select them? Such things weaken the replicability of the scientific studies. In research studies which use machine learning methods, details of the modeling process (e.g., software/program implemented in simulation, ranges of trials for the model parameters and their optimal values for the best model) should be clearly provided by the authors to allow for a reproduction and validation (Wu et al. 2014).

**Table 1:** The parameter settings for GA-ANN (obtained from Wu and Zhou 2022)

Parameter	Setting
Number of neurons in input layer	12
Number of neurons in hidden layer	8
Number of neurons in output layer	1
Training function	trainlm
Loss function	mse
Maxgen	100
Sizepop	30

Wu and Zhou (2022) attempted to compare their models with literature (Nguyen et al. 2022) using RMSE, MAE and MAPE. However, such statistics are highly dependent on data range and data length and range and it is difficult to use them for direct comparison of the two different studies. To be able to compare two studies using RMSE, MAE or MAPE, data split ratio should be the same. The discussers checked the study of Nguyen et al. (2022) and saw that they applied tenfold cross-validation which means that the testing data are not comparable to those of Wu and Zhou (2022). Furthermore, the authors (Nguyen et al. 2022) mentioned some missing data and that they filled missing parts with the mean of the available data. On the other hand, the standard deviations of data provided by Nguyen et al. (2022) are not same as of data provided by Wu and Zhou (2022). This needs further clarification.

Wu and Zhou (2022) state that they used 80% of the whole data set for training and 20% for testing in the first paragraph of the results and discussion section. However, they also write that the GS-SVR model was trained using tenfold cross-validation. The discussers could not understand the methodology followed by the authors. Does this mean that the GA-ANN and GS-SVR models were not trained following the same data split rule? What do you mean by tenfold cross-validation? Did you split the entire data set into 10 folds and tested the models with each part (or fold)? If yes, in this case, testing data set will not be same than that of GA-ANN in which 20% was used for testing. Why didn’t you apply tenfold cross-validation for GA-ANN and ANN also? This point also needs better clarification.

In the abstract of Wu and Zhou (2022), the authors write `*the optimized models were used to train and test the data set*, …`. This statement is not correct because the optimized models cannot be used to train and test data sets. Training set is used to optimize models and then the obtained models are tested and evaluated using testing data set based on selected evaluation metrics. Again in the same section, they say `… *and contribution of these input variables on the output* …` but input variables were not mentioned before. An expansion has not been provided for the GA-ANN and GS-SVR in the text (neither in abstract nor in introduction and other sections). I could understand the meaning after carefully reading research methodology section. In the 4^th^ paragraph of the introduction section, the authors define the ANN, extreme learning machine (ELM), SVR, decision tree, random forest, gradient boosting and adaptive boosting as machine learning (ML) algorithms. This is also not correct because all these are ML methods and training algorithms are used to obtain their model parameters. Such wrong statements are also seen in the last paragraph of the introduction section (`… *with machine learning algorithms*… … *Section 2 describes the two machine learning optimization algorithms GA-ANN and GS-SVR*…).

In the caption of Fig. 6, the authors mention the rough and fine selection but they did not give any information for these in the text. Which methods did they use for these selections? There is no equation and reference for the GA-ANN definition part while GS-SVR has several equations and references. It would be better to calibrate both SVR and ANN using same algorithm, GA or GS. The other issue worth to mention here is that the abbreviations of the input parameters could be used in Table 1, Fig. 4 and whole text instead of *x*_*1*_, *x*_*2*_, …, *x*_*12*_ to be able to better follow the difference between each parameter.

## References

[CR1] Nguyen MST, Trinh MC, Kim SE (2022). Uncertainty quantification of ultimate compressive strength of CCFST columns using hybrid machine learning model. Eng Comput.

[CR2] Wu W, Dandy GC, Maier HR (2014). Protocol for developing ANN models and its application to the assessment of the quality of the ANN model development process in drinking water quality modelling. Environ Model Softw.

[CR3] Wu Y, Zhou Y (2022). Splitting tensile strength prediction of sustainable high-performance concrete using machine learning techniques. Environ Sci Pollut Res.

